# Assessing the efficacy of the ESC 2025 guidelines for the management of cardiovascular disease and pregnancy

**DOI:** 10.1016/j.clinsp.2026.101177

**Published:** 2026-09-30

**Authors:** Akos Koller, Johanna Takács

**Affiliations:** a Department of Morphology and Physiology, Faculty of Health Sciences, Semmelweis University, Budapest, Hungary; b Research Center for Sports Physiology, Hungarian University of Sports Science, Budapest, Hungary; c Institute of Clinical Pathophysiology, Faculty of Medicine, and HUN-REN-SE Cerebrovascular and Neurocognitive Disease Research Group, Semmelweis University, Budapest, Hungary; d Department of Cell and Molecular Physiology, New York Medical College, Valhalla, USA; e Department of Social Sciences, Faculty of Health Sciences, Semmelweis University, Budapest, Hungary

**Keywords:** Preeclampsia, Quality of evidence, Certainty, Uncertainty, Certainty Index

## Abstract

**Objectives:**

Human Pregnancy-related Cardiovascular Diseases (CVDs) are unique and difficult to study because of many unknown, potentially harmful effects of medical interventions on the fetus. Because of that, there are only sparse data and mechanisms available, which could provide recommendations with strong evidence for physicians.

**Study design:**

To assess the efficacy of the European Society of Cardiology (ESC) 2025 Guidelines for the management of cardiovascular disease and Pregnancy (Pregnancy-GL-2025), we used a previously developed mathematical analytic method.

**Main outcome measures:**

A total of 208 Recommendations and Evidence were analyzed, of which 161 Recommendations concern certain CVD. To assess the efficacy, the Certainty/Uncertainty ratio and the Certainty Index (CI, ranging from -1 to +1) were calculated.

**Results:**

In total, the Pregnancy-GL-2025 showed 64.4% of the Class I and III Recommendations (CVDs, 62.7%). The ESC Pregnancy-GL-2025 includes mostly Evidence C (89.4%) and showed 78.8% Certainty (CI = 0.58). Although it includes only a few higher-quality Evidence, these are related to the Class I Recommendations, resulting in a high level of Certainty.

**Conclusions:**

The mathematical analysis of the ESC Pregnancy-GL-2025 showed that it has several helpful Recommendations, yet they are supported primarily by Evidence C. However, because of a few higher-quality evidence related to Class I Recommendations, the overall efficacy of the guideline was relatively high. In addition, the mathematical analysis used instead of frequency analyses does not imply a lower efficacy of a guideline. Thus, we propose that this type of evaluation of guidelines, as a complementary quantitative approach to describing guidelines’ recommendations, can facilitate the development of new guidelines.

## HIGHLIGHTS

• Guidelines help to make decisions regarding the diagnosis and treatment of diseases.
• Should we know how much help is provided by the guidelines to make decisions?.
• A mathematical analysis can objectively measure the help provided by guidelines.
• Pregnancy-GL-2025 has several Recommendations, Class I (yes) and III (no).
• Yet, because of the high number of Evidence C, the help is somewhat limited.

## Introduction

Although human pregnancy is a complex condition, in most cases, there are no complications. Nevertheless, about 6%–8% of pregnancies are considered high-risk due to underlying health problems of the mother and/or fetus, most prominently with cardiovascular diseases (overall CVDs, 1%‒4%; hypertensive disorders with an incidence of 5% to 10%).^1^, ^2^, ^3^, ^4^, ^5^ Because any intervention, diagnostic or treatment, may endanger the foetus, careful consideration should be given to any conditions before applying.^6^

Medical societies develop guidelines, which help physicians and health care workers to make educated decisions regarding diagnostic procedures and treatment modalities in various health conditions. Objective evaluation of the efficacy of clinical guidelines can contribute to the scientific discourse and can enhance the clarity, transparency, and clinical applicability of GLs. In general, GLs consist of Recommendations (I, IIa, IIb, and III) and Evidence (A, B, C) to help decision-making.

One such GL is the 2025 ESC Guidelines for the management of cardiovascular disease and Pregnancy (Pregnancy-GL-2025).^7^ However, Pregnancy-related Cardiovascular Diseases (CVDs) in humans present unique challenges (e.g., hypertension and preeclampsia), making it difficult to conduct appropriate clinical trials that generate high-level evidence to support recommendations. Thus, it is important to reveal the efficacy (the help provided) of Pregnancy-GL-2025, regarding Recommendations and Evidence in Pregnancy-related diseases/in the management of cardiovascular diseases during Pregnancy.

We have used a previously developed mathematical analytic method to assess the efficacy of various Guidelines (GLs) and found that the help provided is not evenly distributed in various disease conditions.^8^, ^9^, ^10^ In the present study, we used this previously developed method to objectively assess the efficacy of Pregnancy-GL-2025 and hypothesized that because of the complexity and uncertainty of pregnancies, the level of Evidence supporting the Recommendations is low.

## Methods

The ESC Pregnancy-GL-2025.^7^was analyzed, which includes a total of 208 Recommendations. Of these, 161 Recommendations concern certain Cardiovascular Diseases (CVDs). For descriptive analysis, the frequency of Classes of Recommendations and Level of Evidence, as well as the frequency of the Level of Evidence in each Class of Recommendations, was examined. To assess the efficacy of the Pregnancy-GL-2025, we used a previously developed mathematical method, 8, 9, 10 which calculates the ratio of Certainty/Uncertainty. Based on the ratio of Certainty/Uncertainty, a Certainty Index was formulated to compare the efficacy of the Pregnancy-GL-2025 in the case of different Cardiovascular Diseases. The Certainty Index ranges from −1 to +1; −1 implies 100% Uncertainty, +1 implies 100% Certainty, and 0 denotes 50%‒50% of Uncertainty and Certainty. The illustration of the calculation of the Certainty Index is shown in Supplementary Figure 1.

## Results

### Frequency of classes of recommendations in the Pregnancy-GL-2025

The ESC Pregnancy-GL-2025 shows a high rate, 64.4%, of Class I (is recommended/is indicated, to do’) and Class III (is not recommended, not to do’) recommendations. Cardiovascular Diseases also include a high rate of these Classes of Recommendations (62.7%) (Table 1). The frequency of Classes of Recommendations in Cardiovascular Diseases see Supplementary Table 1.

**Table 1:** Frequency of classes of recommendations in the ESC Pregnancy-GL-2025 and cardiovascular diseases.

	**Pregnancy-GL-2025** **(n=208)**	**Cardiovascular Diseases** **(n=161)**
	**% (n)**	**% (n)**
**Class I** ‒ Is recommended/ is indicated	60.1(125)	59.0(95)
**Class IIa** ‒ Should be considered	23.1(48)	24.2(39)
**Class IIb** ‒ May be considered	12.5(26)	13.0(21)
**Class III** ‒ Is not recommended	4.3(9)	3.7(6)

### Frequency of level of evidence in the ESC Pregnancy-GL-2025

The Pregnancy-GL-2025 includes primarily Evidence C (89.4%, Table 2). In Cardiovascular diseases, there are 17 Recommendations with Evidence A (n = 1) and Evidence B (n = 16), which are related to certain Cardiovascular Diseases (Supplementary Table 2). For the detailed recommendations with Evidence A and B, see Supplementary Table 3.

**Table 2:** Frequency of the level of evidence in the ESC Pregnancy-GL-2025 and cardiovascular diseases.

	**Pregnancy-GL-2025** **(n=208)**	**Cardiovascular Diseases** **(n=161)**
	**% (n)**	**% (n)**
**Evidence A** ‒ Multiple randomized clinical trials or meta-analyses	0.5 (1)	0.6 (1)
**Evidence B** ‒ Single randomized clinical trials or large non-randomized studies	10.1(21)	10.0(16)
**Evidence C** ‒ Experts and/or small studies, retrospective studies, registries	89.4 (186)	89.4 (144)

### Frequency of level of evidence in each class of recommendations in the ESC Pregnancy-GL-2025

The Recommendation with Evidence A is in Class I, “to do”, and Evidence B is mostly in Class I, “to do”, and in Class IIb, “may do” (Fig. 1A‒B).

**Fig. 1:**
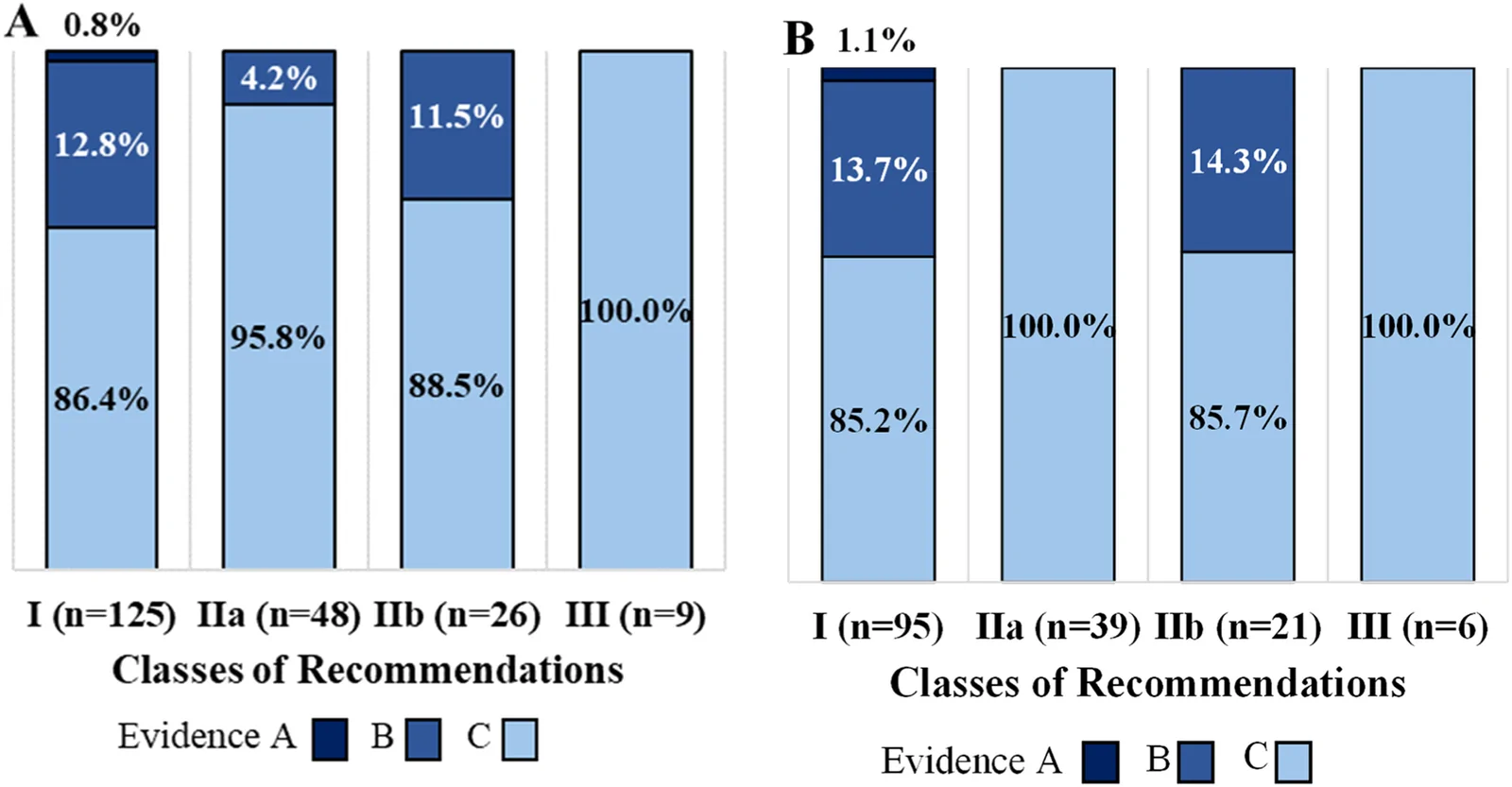
Frequency of the level of evidence in each class of recommendations in the ESC Pregnancy-GL-2025 (A) and cardiovascular diseases (B).

### Certainty/Uncertainty ratio and certainty index in the ESC Pregnancy-GL-2025

The Pregnancy-GL-2025 showed a 0.58 Certainty Index, i.e., 78.8% Certainty. Cardiovascular Diseases showed a 0.61 Certainty Index, i.e., 80.8% Certainty. Even though the Pregnancy-GL-2025 includes only a few pieces of higher-quality Evidence (such as A and B), these pieces of Evidence are related to the ‘to do’ (Class I) recommendations. Importantly, this fact resulted in a high Certainty (Fig. 2). For the detailed Certainty Index in the Cardiovascular Diseases, see Supplementary Figure 2.

**Fig. 2:**
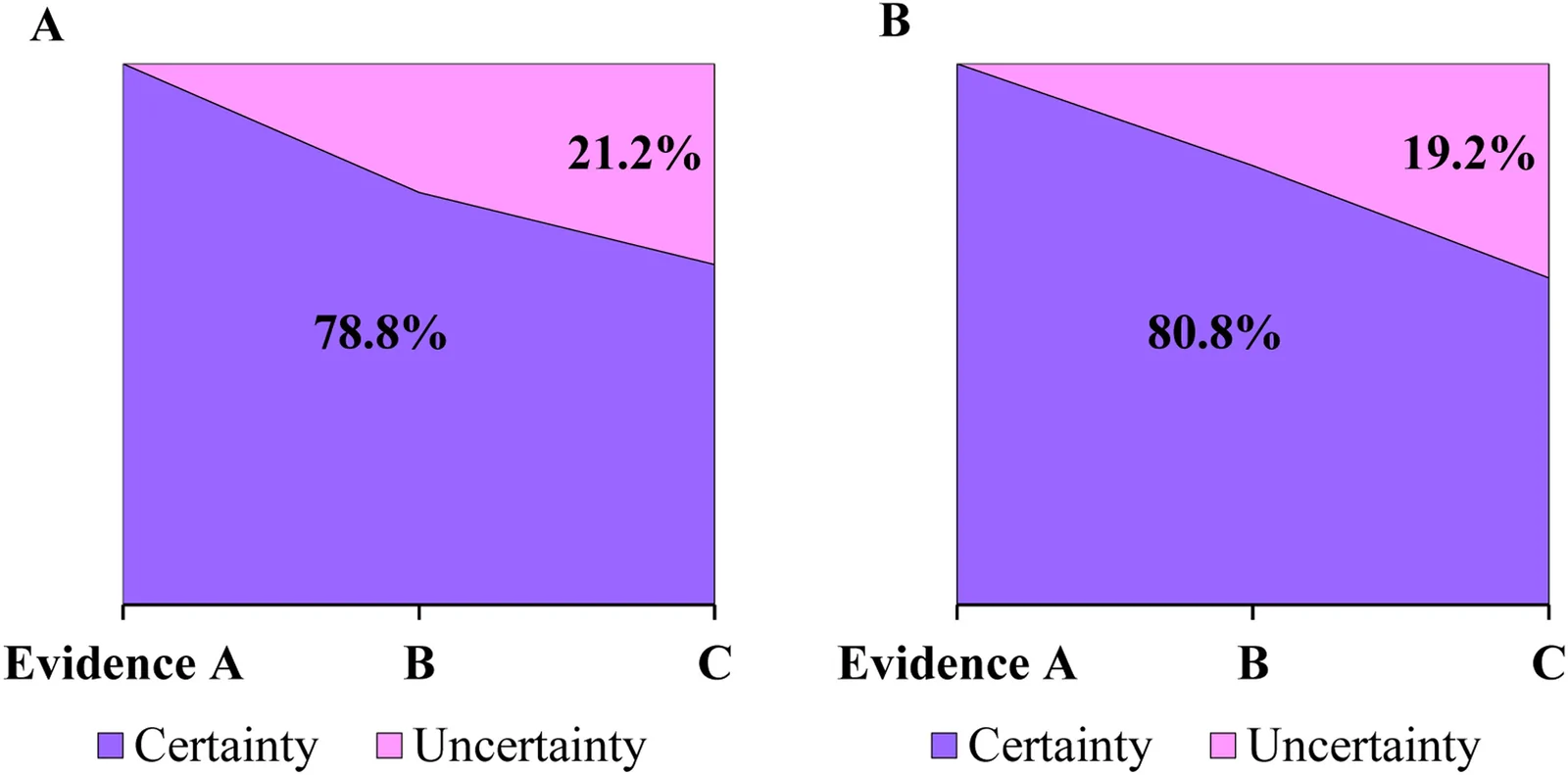
Certainty/Uncertainty ratio in the ESC Pregnancy-GL-2025 (A) and Cardiovascular Diseases (B).

## Discussion

The present study provides a quantitative analysis of the ESC Pregnancy-GL-2025, showing that it contains several useful Recommendations, yet these are supported primarily by Evidence C. At the same time, because a few higher-quality pieces of evidence are related to Class I (to do) Recommendations, the overall efficacy of the guideline was relatively high, as indicated by the Certainty Index. Based on the calculated Certainty Index of certain CVDs, future prospective studies should be prioritized on aortopathies, cardiomyopathies and arrhythmias during Pregnancy to provide novel pieces of higher level of Evidence and Recommendations I and III, improving thereby the efficacy of the Guidelines for the management of cardiovascular disease and Pregnancy.

### Medical guidelines

Scientific medical societies publish guidelines, which can facilitate the decision of physicians and health care workers in various diseases and conditions to make diagnoses and treatments. This is especially important in complex situations, such as cardiovascular diseases in Pregnancy, aiming to standardize the care and to be sure that patients with similar conditions receive similar, high-quality treatment, regardless of geographic and/or social conditions/states.^5^^,^^6^ Guidelines summarize the results of best available human investigations and expert consensus on diagnosis, management, and prevention of diseases, providing an evidence-based decision-making platform. Thereby, guidelines improve safety and outcomes because following guidelines reduce errors, complications, and unnecessary interventions (primum nil nocere: first do no harm). In addition, guidelines help prioritize diagnostic tests and treatments that are proven to work and avoid wasteful or harmful ones, thus promoting the efficient use of resources. Thus, the aim of the present study was to analyze the latest ESC Pregnancy-GL-2025 regarding its efficacy, using a previously developed mathematical method, which was used in the analysis of other GLs.^8^, ^9^, ^10^

### Recommendations and evidence in the ESC Pregnancy-GL-2025

Table 1 and Supplementary Table 1 collected the Recommendations for various Cardiovascular Diseases during Pregnancy. It shows that Class I (recommended/indicated) was relatively high (62.7%) on average; yet in nearly all CVDs, there is only Evidence C behind the Recommendations. In Fig. 1, the frequency of Level of Evidence in each Class of Recommendations in the Pregnancy-GL-2025 (A) and Cardiovascular Diseases (B) is shown.

More detailed analysis regarding Recommendations and Evidence can be found in the Supplementary Files. The analysis indicates that perhaps the most important improvement of Pregnancy-GL-2025 would be necessary in pulmonary and cardiac diseases during Pregnancy, to provide a higher level of Evidence to support the Recommendations. The Pregnancy-GL-2025 may also reflect that findings of some of the animal studies modelling Pregnancy-related diseases cannot be translated directly to human conditions.^11^, ^12^, ^13^, ^14^, ^15^, ^16^, ^17^ Moreover, guidelines can likely be improved by including comprehensive reviews regarding certain therapies for hypertensive pregnancies, and other disease conditions related to Pregnancy.^18^ Thus, in the future, improved preclinical studies, experimental models, ^19^ development of novel biomarkers, 20, 21, 22, 23, 24 wearable devices (smartwatch, smart rings, which can track many physiological parameters, sleep quality and stress) and artificial intelligence approaches^25^, ^26^, ^27^ may be need to be included in the study design for a better outcome of clinical trials, which can provide higher level of Evidence for the given Recommendations.

### Certainty/Uncertainty ratio (C/UC) and the formulated certainty index (CI) in the ESC Pregnancy-GL-2025

After analyzing the frequency of Recommendations and Evidence, we calculated the Certainty/Uncertainty ratio and Certainty Index, which are shown in Fig. 2. The Certainty/Uncertainty ratio (C/UC) and the formulated Certainty Index (CI) ranged from −1 to +1; −1 implies 100% Uncertainty, +1 implies 100% Certainty, and 0 denotes 50%‒50% of Uncertainty and Certainty. On average, the Pregnancy-GL-2025 showed a 0.58 Certainty Index, i.e., 78.8% Certainty. Cardiovascular Diseases showed a 0.61 Certainty Index, i.e., 80.8% Certainty. This is because a few of the higher-quality evidence, Evidence A and B, were in the ‘to do’ (Class I) recommendations.

### Limitations of the present study

The mathematical method used in the present study resulted in a guideline-based certainty metric, i.e., the Certainty Index, without evaluating the clinical reasoning and practice, context or nuance behind the recommendations. Thus, the results of the method serve as a ‘quality control’ of guidelines but not a professional or clinical evaluation or critique of them. The developed Certainty Index needs further external validation and exploration of clinical interpretability. Nevertheless, the results can be useful in providing information about issues to be clarified and investigated in future studies to increase the efficacy of guidelines. A further important limitation of this method involves the fact that any inherent biases in guideline development, like expert-driven judgments, selective evidence use, or methodological constraints, are transferred into our results because the method is based on the grading system applied in the underlying clinical practice guideline. Finally, it should be emphasized that there are no systematic methods beyond simple frequency analyses which can measure the efficacy of guidelines; consequently, the method used in this study will probably be enhanced and refined in subsequent clinical studies.

### The significance of the mathematical analysis used

Using the presented mathematical analysis, a guideline-based certainty metric, i.e., the Certainty Index, can be calculated, which is derived from a structured grading system applied in medical guidelines. The two main purposes of the present study were 1) To use the previously developed method to assess the efficacy of this guideline, because at present there are no systematic methods to do that; and 2) To test the applicability of this analytic method on a guideline known to contain primarily Evidence C, in contrast to other guidelines analyzed previously.^8^, ^9^, ^10^

Previous studies evaluating different guidelines and comparing the improvement of guidelines over time concluded that the vast majority of recommendations are supported by a lower quality of Evidence,^28^, ^29^, ^30^, ^31^, ^32^, ^33^ and demonstrated a consistent temporal trend in the improvement of the levels of Evidence.^28^^,^^30^, ^31^, ^32^^,^^34^

However, based on a method that analyses only the frequency of Recommendations and Evidence, it can be concluded that a high proportion of recommendations are based on a lower quality of evidence. Thus, the results of this method can question the efficacy of a guideline and can decrease the implementation of the recommendations included in a guideline to clinical practice.^28^, ^29^, ^30^, ^31^, ^32^, ^33^, ^34^, ^35^, ^36^

It is important to note that the Classes of Recommendations and Level of Evidence are assigned separately; thus, the level of Evidence does not necessarily affect the class of the Recommendations. One may assume that a higher level of Evidence directly correlates with a stronger clinical Recommendation.^34^, ^35^, ^36^ Classes of Recommendations are based on benefit-risk balance, clinical relevance, and consensus. Level of Evidence based on study design and data quality. Thus, strong Recommendations may be issued, even if the evidence is limited. For example, when benefits clearly outweigh harms, clinical experience is extensive, ethical or practical constraints limit randomized trials, or the intervention is life-saving or time-critical. Guidelines provide Recommendations based on the best available Evidence, even if the evidence is only of low quality.^37^^,^^38^ This should not reduce the efficacy of a guideline if an adequate objective analysis is applied. This is particularly important in ESC Pregnancy-GL-2025, where a higher level of Evidence is not available because randomized trials cannot be conducted for ethical or practical reasons.

Previous studies showed^8^, ^9^, ^10^ that an analysis, which is based only on the frequency of the Classes of Recommendations and/or Level of Evidence (Class by Level or Level by Class), resulted in biased frequency data and conclusions. For example, “comparison of the helpfulness of ESH 2013, 2018, and 2023 guidelines” for the management of arterial hypertension ^8^ based on the frequency data, it might be concluded that, rather than leading to improvements over the past ten years, the latest guidelines contain weaker Recommendations and a lower level of Evidence, even though the number of Recommendations has doubled. Yet, the Certainty Index clearly showed the improvement of GL over time.

The mathematical analysis developed^8^, ^9^, ^10^ can identify specific areas within a guideline that require improvement; it can compare the efficacy of different parts, diseases and treatments/interventions, and certain guidelines. At the same time, in line with the results of our and previous studies, it may be beneficial during the development of guidelines to manage more effectively the limitations of rigid classifications based on categorized systems like the GRADE framework, AAP or ESC Guidelines Classifications Scheme.^39^, ^40^, ^41^ Thus, it can be recommended to apply weighting based on professional experience and consensus to a lower level of Evidence to reflect real-world clinical situations.

## Conclusions

The mathematical analysis of the ESC Pregnancy-GL-2025 shows that it contains several useful Recommendations, yet these are supported predominantly by Evidence Level C. At the same time, because of a few of the higher-quality evidence related to Class I (to do) Recommendations, the overall efficacy of the guideline was relatively high, as indicated by the Certainty Index. In addition, the mathematical analysis used instead of frequency analyses does not imply a lower efficacy of a guideline. Thus, we propose that this type of evaluation of clinical guidelines, as a complementary quantitative approach to describing guidelines’ recommendations, can contribute to an assessment of their efficacy and facilitate the development of new guidelines.

## Ethics approval and consent to participate

NA.

## Consent for publication

NA.

## Declaration of generative AI and AI-assisted technologies in the writing process

The authors declare that no generative AI or AI-assisted technologies were used in the writing of this manuscript.

## Authors’ contributions

Akos Koller: Conceptualization, writing-original draft, writing-review & editing, supervision.

Johanna Takács: Writing-original draft, writing-review & editing, formal analysis. all gave final approval and agreed to be accountable for all aspects of work, ensuring integrity and accuracy.

## Funding

Ministry for Innovation and Technology, Hungary, National Research, Development and Innovation Fund, OTKA K 132,596 (AK), TKP2021-EGA-37 (AK), TKP2021-EGA-25 (JT), EKÖP-2024–151 (JT); Hungarian Academy of Sciences Post-Covid 2021–2024 (AK and JT), Hungarian Hypertension Society, HHS-2025 Research Grant (JT and AK).

## Data availability

The ESC Pregnancy-GL-2025 is available at the European Heart Journal. 2025;46:4462–568. doi: 10.1093/eurheartj/ehaf193.

## Declaration of competing interest

The authors declare no conflicts of interest.

## Footnotes

Supplementary material associated with this article can be found, in the online version, at doi:10.1016/j.clinsp.2026.101177.
