# Supplementary material for: Assessing the efficacy of the ESC 2025 guidelines for the management of cardiovascular disease and pregnancy
Source: Clinics (Sao Paulo). 2026 Sep 30;81:101177. doi: 10.1016/j.clinsp.2026.101177 (PMC13657149; doi:10.1016/j.clinsp.2026.101177)
Supplement: Supplementary file 1 [file mmc1.docx]

**CLINICS-D-26-01368_Supplementary Materials**

**Supplementary Figure 1**. Forming the Certainty Index (Koller, Takács, 2021, 2024)

**Supplementary Table 1.** The Frequency of Classes of Recommendations in Cardiovascular Diseases

**Supplementary Table 2**. The Frequency of the Level of Evidence in Cardiovascular Diseases

**Supplementary Figure 2.** Certainty/Uncertainty ratio in the Cardiovascular diseases

**Supplementary Table 3.** Detailed recommendations with Evidence A and B in the Pregnancy-GL-2025 for the management of cardiovascular diseases during pregnancy [7].

**Supplementary Figure 1**. Forming the Certainty Index (Koller, Takács, 2021, 2024)

1. Collapsing Classes of Recommendations into two categories: to define certainty and uncertainty, we collapsed the Classes of Recommendations into two categories: uncertainty (‘should + may do’) and certainty (‘to do + not to do’). 2. Calculation LEVEL areas under CLASS: we calculated the areas using the trapezoid area formula: $=\left( a+b \right)\frac{h}{2}$, where ‘a’ and ‘b’ are the bases (parallel sides) and ‘h’ is the height of the trapezoid. 3. Defining certainty and uncertainty rate: Based on the calculation in step 2, we expressed trapezoid areas in percentages. 4. Forming certainty index: Based on the percentages in step 3, an index of certainty/uncertainty was formed, which ranges from −1 to +1; a value of −1 implies 100% uncertainty, a value of +1 implies 100% certainty, and a value of 0 implies fifty–fifty certainty and uncertainty. The depicted certainty indexes (CI = −0.76 and CI = +0.58) are random examples showing the meaning of a certainty index with the rate of the percentages of certainty and uncertainty.

References:

Koller A, Takács J. A Guideline for Guidelines: A Novel Method to Assess the Helpfulness of Medical Guidelines. Journal of Clinical Medicine. 2024;13:3783. doi: 10.3390/jcm13133783

Koller A, Takács J. Translation of scientific evidence into cardiovascular guidelines. JBI Evidence Implementation. 2021;19:437-445. doi: 10.1097/XEB.0000000000000266

**Supplementary Table 1.** The Frequency of Classes of Recommendations in Cardiovascular Diseases

|  | **Aortopathies** | **Arrhythmias** | **Valvular heart disease** | **Primary arrhythmia syndromes** |
| --- | --- | --- | --- | --- |
|  | **(n=35)** | **(n=27)** | **(n=20)** | **(n=14)** |
|  | **%(n)** | **%(n)** | **%(n)** | **%(n)** |
| **Class I** - Is recommended/ is indicated | 57.1(20) | 55.6(15) | 65.0(13) | 50.0(7) |
| **Class IIa** - Should be considered | 22.9(8) | 25.9(7) | 20.0(4) | 50.0(7) |
| **Class IIb** - May be considered | 17.1(6) | 14.8(4) | 10.0(2) | 0.0(0) |
| **Class III** - Is not recommended | 2.9(1) | 3.7(1) | 5.0(1) | 0.0(0) |
|  | **Hypertensive disorders** | **Cardiomyo-pathies** | **Heart failure** | **Venous tromboembolism** |
|  | **(n=13)** | **(n=12)** | **(n=11)** | **(n=7)** |
|  | **%(n)** | **%(n)** | **%(n)** | **%(n)** |
| **Class I** - Is recommended/ is indicated | 84.6(11) | 50.0(6) | 54.5(6) | 57.1(4) |
| **Class IIa** - Should be considered | 7.7(1) | 25.0(3) | 18.2(2) | 28.6(2) |
| **Class IIb** - May be considered | 7.7(1) | 16.7(2) | 18.2(2) | 14.3(1) |
| **Class III** - Is not recommended | 0.0(0) | 8.3(1) | 9.1(1) | 0.0(0) |
|  | **Coronary artery disease** | **Peripartum cardio-myopathy** | **Pulmonary arterial hypertension** | **Congenital heart disease** |
|  | **(n=7)** | **(n=6)** | **(n=5)** | **(n=4)** |
|  | **%(n)** | **%(n)** | **%(n)** | **%(n)** |
| **Class I** - Is recommended/ is indicated | 71.4(5) | 16.7(1) | 60.0(3) | 100.0(4) |
| **Class IIa** - Should be considered | 14.3(1) | 50.0(3) | 20.0(1) | 0.0(0) |
| **Class IIb** - May be considered | 14.3(1) | 33.3(2) | 0.0(0) | 0.0(0) |
| **Class III** - Is not recommended | 0.0(0) | 0.0(0) | 20.0(1) | 0.0(0) |

**Supplementary Table 2**. The Frequency of the Level of Evidence in Cardiovascular Diseases

|  | **Aortopathies** | **Arrhythmias** | **Valvular heart disease** | **Primary arrhythmia syndromes** |
| --- | --- | --- | --- | --- |
|  | **(n=35)** | **(n=27)** | **(n=20)** | **(n=14)** |
|  | **%(n)** | **%(n)** | **%(n)** | **%(n)** |
| **Evidence A** | **0.0(0)** | **0.0(0)** | **0.0(0)** | **0.0(0)** |
| **Evidence B** | **0.0(0)** | **0.0(0)** | 5.0(1) | 21.4(3) |
| **Evidence C** | 100.0(35) | 100.0(27) | 95.0(19) | 78.6(11) |
|  | **Hypertensive disorders** | **Cardiomyo-pathies** | **Heart failure** | **Venous tromboembolism** |
|  | **(n=13)** | **(n=12)** | **(n=11)** | **(n=7)** |
|  | **%(n)** | **%(n)** | **%(n)** | **%(n)** |
| **Evidence A** | 7.6(1) | **0.0(0)** | **0.0(0)** | **0.0(0)** |
| **Evidence B** | 46.2(6) | **0.0(0)** | 9.1(1) | 28.6(2) |
| **Evidence C** | 42.6(6) | 100.0(12) | 90.9(10) | 71.4(5) |
|  | **Coronary artery disease** | **Peripartum cardiomyopathy** | **Pulmonary arterial hypertension** | **Congenital heart disease** |
|  | **(n=7)** | **(n=6)** | **(n=5)** | **(n=4)** |
|  | **%(n)** | **%(n)** | **%(n)** | **%(n)** |
| **Evidence A** | **0.0(0)** | **0.0(0)** | **0.0(0)** | **0.0(0)** |
| **Evidence B** | **14.3(1)** | **16.7(1)** | **0.0(0)** | **25.0(1)** |
| **Evidence C** | **85.7(6)** | **83.3(5)** | **100.0(5)** | **75.0(3)** |

**Supplementary Figure 2.** Certainty/Uncertainty ratio in the Cardiovascular diseases

**Supplementary Table 3.** Detailed recommendations with Evidence A and B in the Pregnancy-GL-2025 for the management of cardiovascular diseases during pregnancy [7].

| **CARDIOVASCULAR DISEASES** | **RECOMMENDATION** | **CLASS** | **LEVEL** |
| --- | --- | --- | --- |
| **Hypertensive disorders** | **Low-dose aspirin (75–150 mg daily) is recommended in women at moderate or high risk of pre-eclampsia (i.e. at least one high risk factor or two moderate risk factors for pre-eclampsia) from weeks 12 to 36/37.** | **I (to do)** | **A** |
| *Primary arrhythmia syndromes* | Beta-blockers, with pre-pregnancy dose and with nadolol and propranolol as drugs of choice, are recommended during pregnancy in women with LQTS. | **I (to do)** | **B** |
|  | It is recommended to continue beta-blocker therapy during lactation in women with LQTS to reduce arrhythmic risk. | **I (to do)** | **B** |
|  | Pre-pregnancy dose beta-blocker of nadolol or propranolol are recommended in women with LQT2, particularly in the post-partum period, which represents a high-risk period for life-threatening arrhythmias. | **I (to do)** | **B** |
| *Congenital heart disease* | Vaginal delivery is recommended in most women with ACHD. | **I (to do)** | **B** |
| *Venous thromboembolism* | For pregnant or post-partum women at high risk of VTE, a prophylactic fixed dose of LMWH is recommended over a higher weight-adjusted dose to reduce the risk of VTE. | **I (to do)** | **B** |
|  | In pregnant women or women in the post-partum period with suspicion of VTE (DVT and/or PE), an immediate formal diagnostic assessment with validated methods is recommended and should not be postponed. | **I (to do)** | **B** |
| *Coronary artery disease* | Low-dose ASA is recommended as the antiplatelet treatment of choice during pregnancy and lactation when single antiplatelet treatment is indicated. | **I (to do)** | **B** |
| *Hypertensive disorders* | It is recommended to aim for systolic BP <140 mmHg and diastolic BP <90 mmHg in pregnant women. | **I (to do)** | **B** |
|  | In women with gestational hypertension, initiation of drug treatment is recommended at systolic BP ≥140 mmHg or diastolic BP ≥90 mmHg. | **I (to do)** | **B** |
|  | Methyldopa is recommended for the treatment of hypertension in pregnancy. | **I (to do)** | **B** |
|  | In women with pre-eclampsia without severe features, delivery is recommended at 37 weeks. | **I (to do)** | **B** |
|  | In women with gestational hypertension, delivery is recommended at 39 weeks | **I (to do)** | **B** |
| *Prosthetic valves* | A bioprosthetic valve is recommended (over a mechanical valve) in young women contemplating pregnancy requiring a valve prosthesis. | **I (to do)** | **B** |
| *Peripartum cardiomyopathy* | Bromocriptine treatment may be considered in addition to optimal HF treatment to enhance recovery of LV function in women with PPCM | **IIb (may do)** | **B** |
| *Hypertensive disorders* | Home BP monitoring may be considered as an adjunct to office BP measurements in pregnant women to detect new-onset hypertension or for monitoring BP control | **IIb (may do)** | **B** |
| *Heart failure* | Preventing lactation may be considered in women with severe HF due to the high metabolic demands of  lactation. | **IIb (may do)** | **B** |
